# Post-translational modifications of influenza virus proteins

**DOI:** 10.1016/j.gendis.2026.102271

**Published:** 2026-06-02

**Authors:** Menghuan Cui, Shuyi Wang, Xiao Fei, Guijie Guo

**Affiliations:** a Key Laboratory of Animal Pathogen Infection and Immunology of Fujian Province, College of Animal Sciences, Fujian Agriculture and Forestry University, Fuzhou, Fujian 350002, China; b Joint Laboratory of Animal Pathogen Prevention and Control of Fujian-Nepal, College of Animal Sciences, Fujian Agriculture and Forestry University, Fuzhou, Fujian 350002, China; c Engineering Research Center for Animal Breeding and Sustainable Production, College of Animal Sciences, Fujian Agriculture and Forestry University, Fuzhou, Fujian 350002, China

**Keywords:** Influenza viral proteins, Influenza virus, Post-translational modifications (PTMs), SUMOylation, Viral infection

## Abstract

Influenza virus remains a global public health threat, with its infection mechanisms and pathogenicity critically influenced by post-translational modifications (PTMs) of viral proteins. These PTMs play pivotal roles throughout the viral infection cycle by governing the structure, function, and host–cell interactions of viral proteins. While traditional PTMs, such as phosphorylation of the polymerase complex, ubiquitination of viral scaffolds, and glycosylation of surface antigens, remain central, recent multi-omics advances have unveiled a broader landscape, including SUMOylation, acetylation, and carboxylation. Furthermore, these modifications do not function in isolation; they form a multilayered crosstalk network that orchestrates the delicate balance between viral fitness and host immune evasion. These advancements provide crucial foundations for understanding influenza pathogenesis and informing the development of novel antiviral strategies. This review provides a comprehensive overview of the functional roles of both host- and virus-mediated PTMs during the influenza virus infection process. In addition, we highlight current research gaps and suggest future directions for targeting PTMs in therapeutic design. Such insights offer promising avenues for innovative antiviral interventions.

## Introduction

Influenza virus, a member of the *Orthomyxoviridae*, is an enveloped virus whose segmented genome comprises eight distinct negative-sense RNA strands.^1^^,^^2^ Each RNA segment is encapsidated by nucleoprotein (NP) and encodes at least one essential viral protein—including RNA polymerase subunits (PB2, PB1, PA), surface glycoproteins hemagglutinin (HA) and neuraminidase (NA), matrix proteins M1 and M2, and non-structural proteins NS1 and NS2.^3^^,^^4^ The *influenza A virus* life cycle encompasses receptor-mediated entry and nuclear replication of viral ribonucleoproteins (vRNPs), and culminates in assembly, budding, and NA-facilitated release of progeny virions.^5^^,^^6^

Post-translational modifications (PTMs) are covalent alterations involving the attachment of functional groups or proteins to specific amino acid residues.^7^^,^^8^ The study of PTMs has evolved through key discoveries, including protein phosphorylation, disulfide bonds, histone acetylation, and the ubiquitin-proteasome system, which together established the foundation for understanding PTMs as essential regulatory mechanisms.^9^, ^10^, ^11^ Modern research has documented over 700 PTM types in resources like UniProt, with functional roles in signaling and epigenetics being actively expanded by computational predictions.^12^^,^^13^

Common PTMs include ubiquitination, glycosylation, lipidation, methylation, phosphorylation, and acetylation.^14^^,^^15^ These modifications are catalyzed by dedicated enzymes such as ubiquitin E3 ligases, glycosyltransferases, kinases, poly (ADP-ribose) polymerases (PARPs), and acetyltransferases.^16^^,^^17^ During viral infection, PTMs play dual roles: host cells utilize them to restrict viral replication—by activating immune responses and inhibiting viral protein synthesis—while viruses hijack PTM machinery to enhance protein solubility, antigenicity, and virulence, thereby promoting viral fitness.^18^ PTMs modulate influenza virus proteins through multiple mechanisms.^8^^,^^19^ Glycosylation of NA modulates viral pathogenicity: removal of the NA cytoplasmic tail reduces protein expression and stability, altering both pathogenicity and virion morphology.^20^

Host–virus PTM interactions promote immune evasion through multiple pathways, including interference with antigen presentation, modulation of immune checkpoint function, structural alteration of viral proteins to evade antibody recognition, and impairment of immune cell activity.^21^, ^22^, ^23^, ^24^ Studies reveal that virus- or host-mediated PTMs enhance viral replication, assembly, and release while suppressing host immune responses, particularly interferon-stimulated gene (ISG) expression, a cornerstone of antiviral defense.^25^ For instance, NP interacts with mitochondrial antiviral-signaling protein (MAVS) and Toll-interacting protein (TOLLIP), inducing mitophagy-mediated MAVS degradation.^26^^,^^27^ This process suppresses type I interferon signaling and promotes viral replication.^26^

Critically, as mapped in Figure 1, every influenza viral protein serves as a substrate for a distinct yet overlapping set of PTMs. For instance, PA, PB1, and PB2 are hotbeds for phosphorylation and ubiquitination, while the surface glycoproteins HA and NA are dominated by glycosylation.^28^^,^^29^ These modifications highlight the complexity of functional regulation in viral proteins and underscore the universality of PTM networks in viral pathogenesis.^30^ The wide variety of PTMs not only fine-tunes viral protein activity but also plays a pivotal role in host–pathogen interactions, ultimately influencing viral infectivity and immune evasion as mapped in Figure 3, Figure 4.^18^^,^^31^

**Figure 1:**
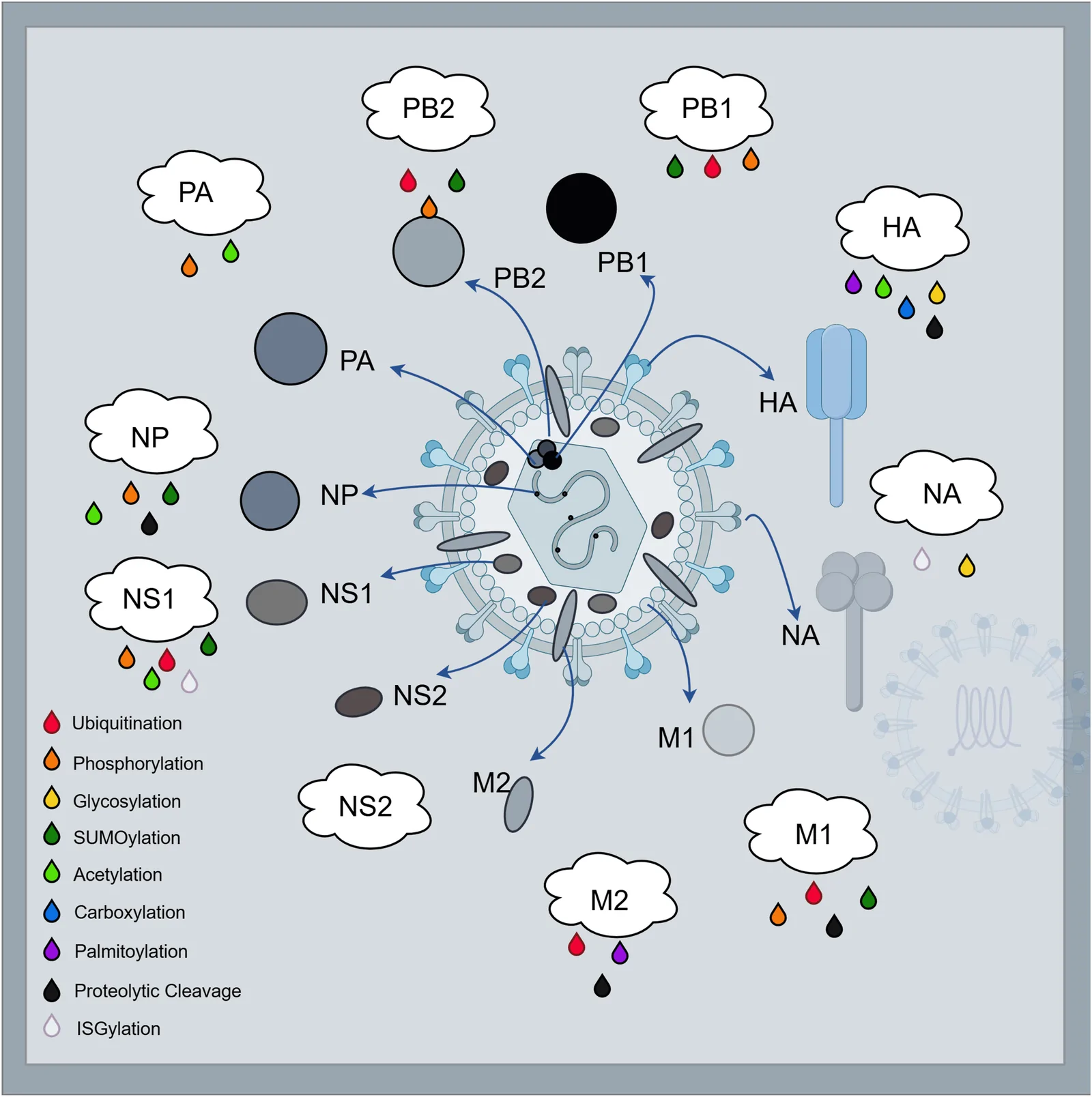
Post-translational modifications (PTMs) of influenza virus proteins. The viral architecture comprises core structural and functional proteins—including polymerase subunits PA, PB1, PB2; surface glycoproteins HA and NA; nucleoprotein NP; matrix protein M1; and non-structural protein NS1—which undergo ubiquitination, phosphorylation, glycosylation, SUMOylation, and other modifications during infection.

**Figure 2:**
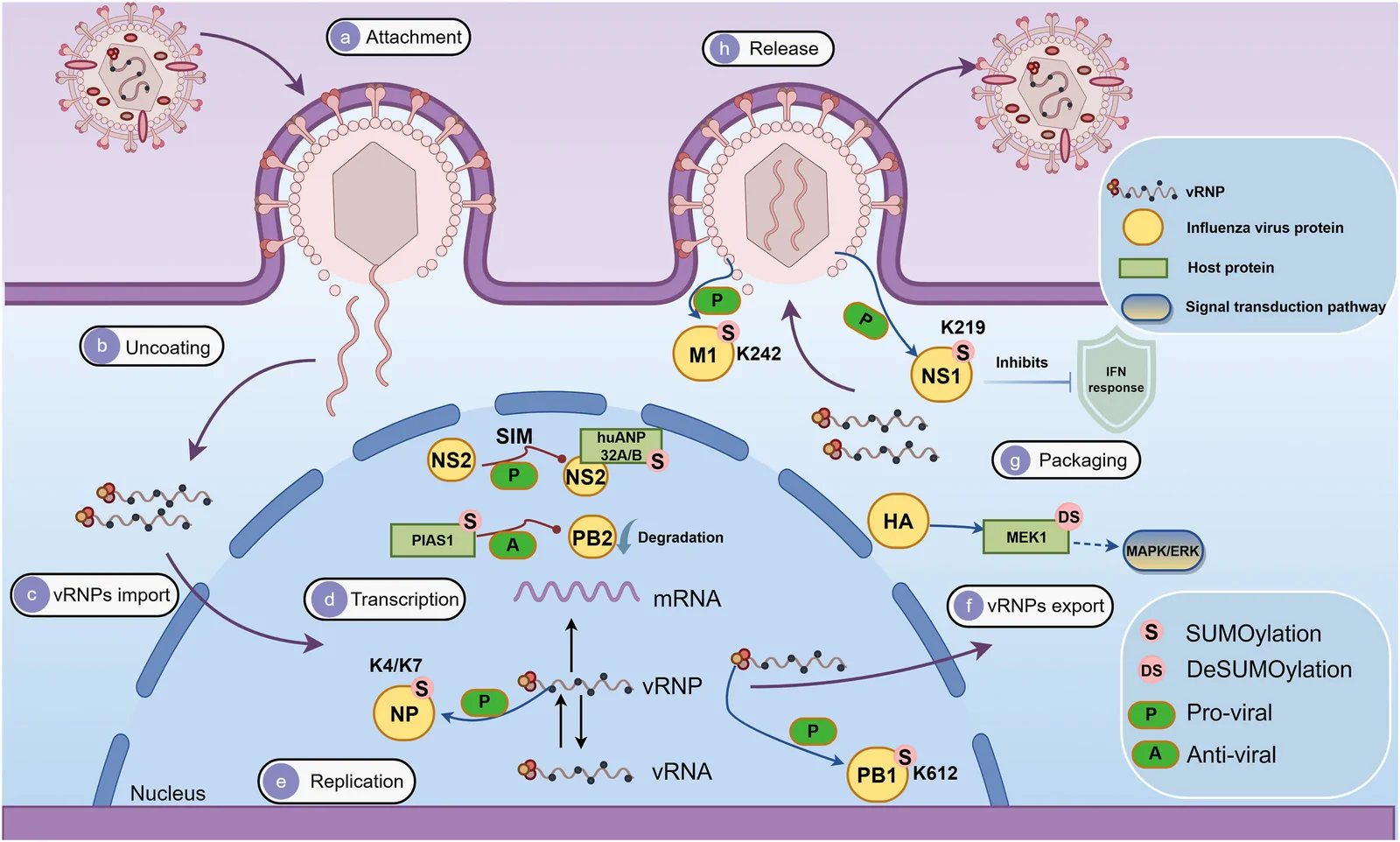
Roles of SUMOylation in the influenza virus life cycle. As outlined in steps a–e, during virus entry and genome replication/transcription, SUMOylation of NP at K4/K7 is essential for its nuclear localization and viral growth.^100^^,^^110^ SUMOylation of PB1 at K612 enhances vRNA binding and polymerase activity.^101^ NS2 binds to SUMOylated huANP32 A/B via its SIM to enhance polymerase activity,^102^^,^^103^ while PIAS1-mediated SUMOylation of PB2 reduces its stability.^104^ In the late stage of the viral life cycle (step f–h), M1 SUMOylation (K242) mediates matrix formation,^105^ and HA triggers the deSUMOylation of MEK1 to promote RNP export via the MAPK/ERK pathway.^107^ For host immune antagonism, SUMOylation of NS1 (K219) stabilizes the protein and suppresses IFN response.^108^^,^^206^

**Figure 3:**
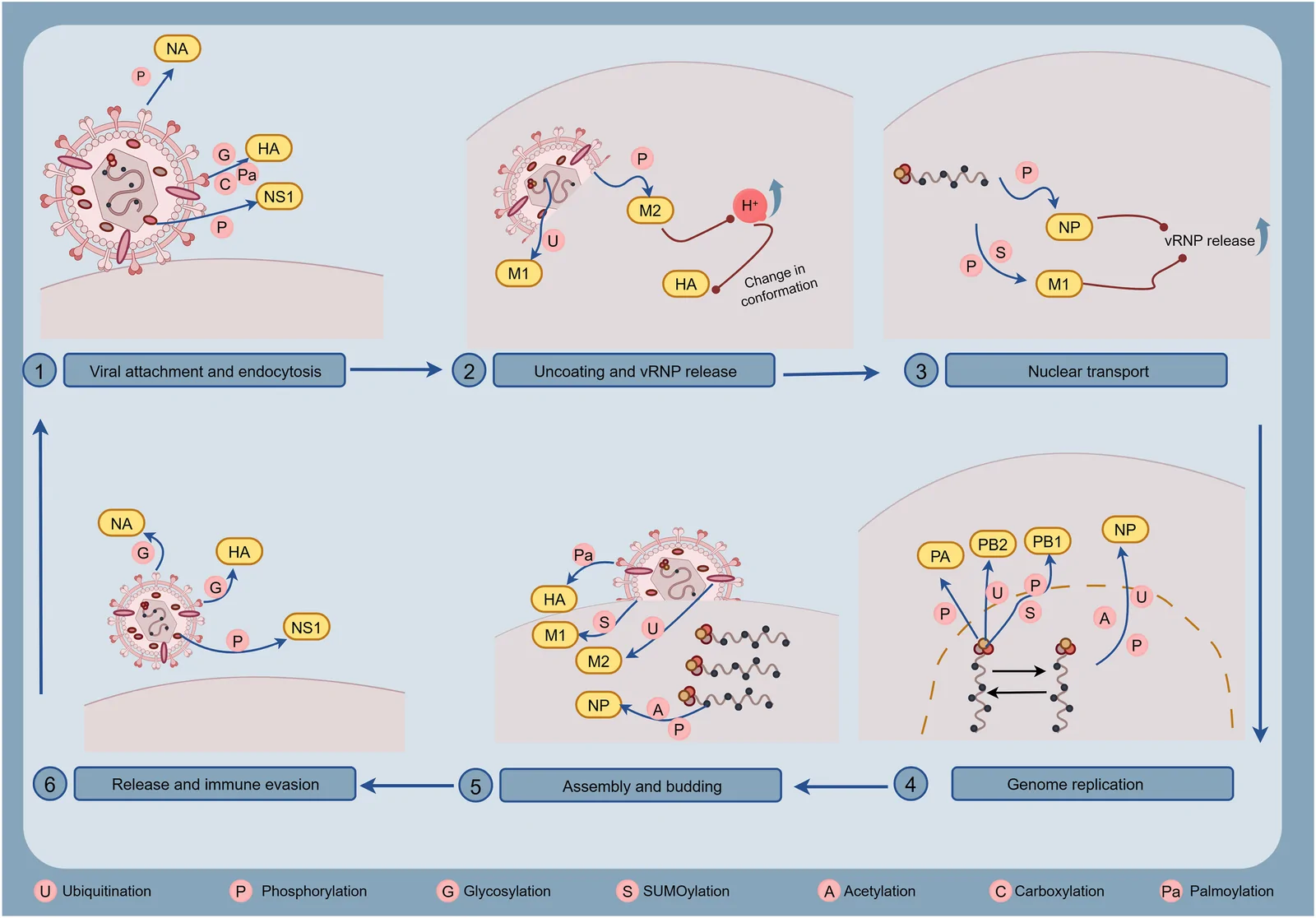
Regulation of the influenza virus life cycle by post-translational modifications. **1)** Viral attachment and endocytosis: NA phosphorylation, HA glycosylation/palmitoylation/E225 carboxylation, and NS1 phosphorylation orchestrate receptor interaction efficiency and initiate endocytosis. **2)** Uncoating and vRNP release: M1 ubiquitination, along with M2 phosphorylation that facilitates endosomal acidification to induce HA conformational changes, regulates membrane fusion and vRNP release during uncoating. **3)** Nuclear transport: M1 SUMOylation stabilizes vRNP complexes while NP phosphorylation regulates their nuclear–cytoplasmic shuttling, coordinately facilitating vRNP release. **4)** Genome replication: Post-translational modifications regulate RNA synthesis by modulating protein stability, enzymatic activity, and complex assembly. **5)** Assembly and budding: M2 ubiquitination, M1 SUMOylation, HA palmitoylation, and NP acetylation ensure efficient viral assembly and budding by coordinating genome packaging, membrane recruitment, and particle release. **6)** Release and immune evasion: On the one hand, the virus actively exploits HA glycosylation to cloak itself; on the other hand, its core immunosuppressive protein NS1 can be counteracted by the host through phosphorylation.

**Figure 4:**
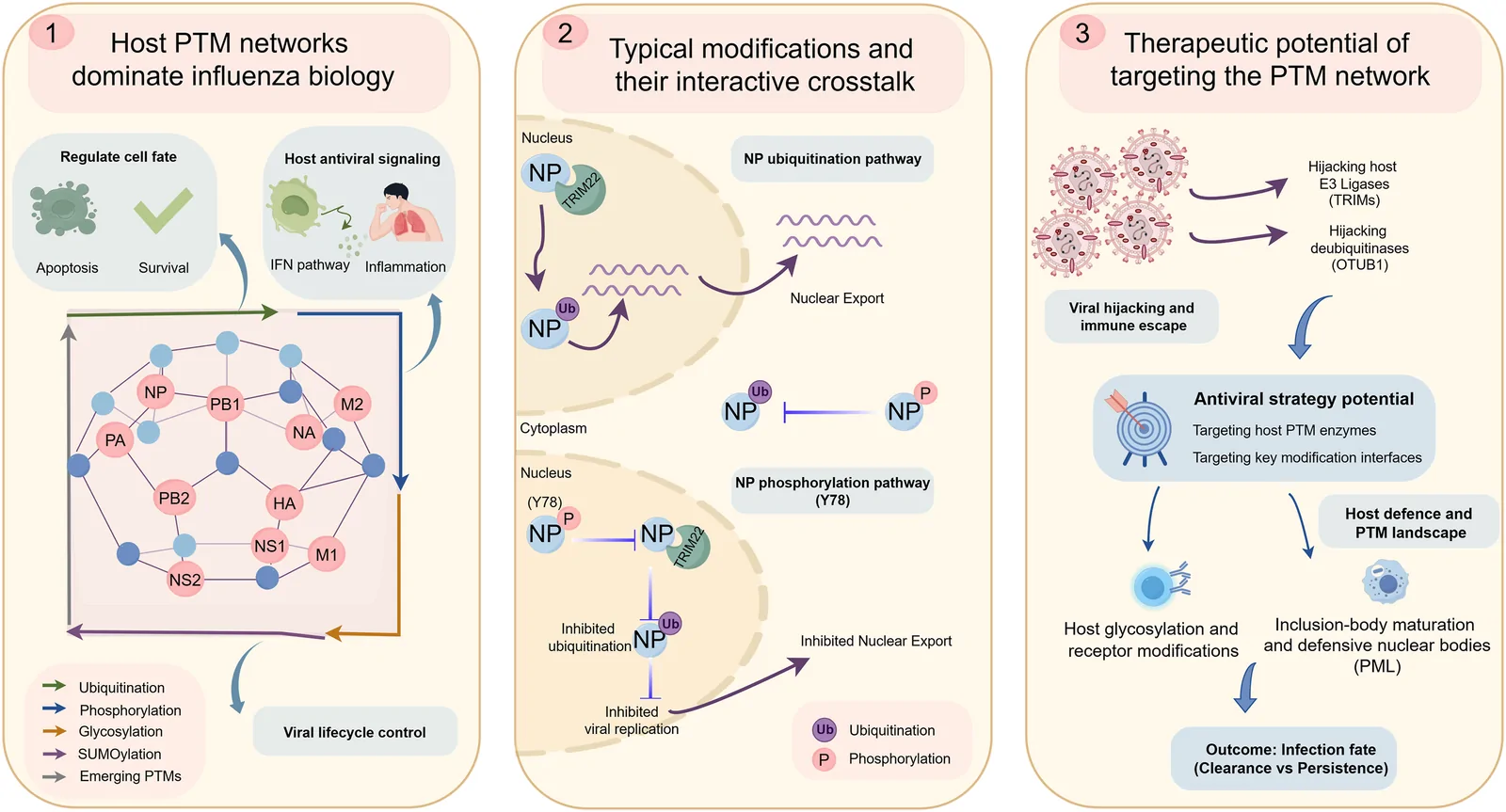
The multilayered post-translational modification (PTM) network in influenza infection. **1)** Host PTM networks dominate influenza biology: Modifications include ubiquitination, phosphorylation, glycosylation, SUMOylation, and other emerging modifications, forming a multilayered, interwoven network that regulates viral components and host antiviral signaling, inflammatory responses, and cell fate.^8^**2)** Typical modifications and their interactive crosstalk: A typical example of PTM crosstalk is the phosphorylation of NP, which suppresses its ubiquitination by disrupting interaction with the E3 ligase TRIM22.^143^**3)** Therapeutic potential of targeting the PTM network: Influenza viruses hijack this network by co-opting host enzymes like E3 ligases and deubiquitinases, thereby suppressing antiviral responses and modulating viral protein stability, assembly, and immune evasion.^8^^,^^207^ Consequently, antiviral strategies are increasingly targeting these host PTM enzymes or their critical interaction interfaces.^208^ This regulatory landscape extends to other host processes, from glycosylation and inclusion-body maturation to PML-nuclear-body defenses.^158^^,^^209^^,^^210^

The pervasive influence of PTMs on the influenza virus life cycle, from entry to egress, has been systematically elucidated, underscoring their role as master regulators of viral replication.^32^ Notably, this regulation extends critically to the interface with the host, where PTMs are pivotal in modulating innate immune responses, creating a complex battleground for virus–host interactions.^8^ The rapid expansion of this field has been propelled by advanced proteomic technologies, enabling the discovery and functional characterization of a vast array of viral and host protein modifications.^30^ To provide a coherent framework for the complex mechanisms to be discussed, Figure 3 delineates the influenza virus life cycle into six critical stages—from attachment to immune evasion—and maps the key regulatory PTMs onto each step. Synthesizing recent findings from the past five years, we will explore how ubiquitination, phosphorylation, glycosylation, the small ubiquitin-like modifier (SUMO), and emerging modifications collectively orchestrate this precisely timed sequence of events, as outlined in this master roadmap, alongside emerging therapeutic strategies targeting these pathways for broad-spectrum antiviral development.

### Major types and mechanisms of PTMs in influenza virus proteins

As summarized in Table 1, PTMs play pivotal roles throughout the viral replication cycle and in mediating host adaptation.

**Table 1:** Post-2018 advances in protein post-translational modifications of influenza virus and their functional implications.

Post-translational modification	Viral protein	Modification site/mechanism	Functional outcome	Pro-/anti-viral	Key subtypes	References (journal, year)
Ubiquitination	M2	M2-K48	Coordinate the efficient packaging of viral genomes into viral particles	Pro-viral	H1N1	*J Virol* (2018)*^43^*
Ubiquitination	NP	TRIM41-mediated ubiquitination and degradation	Limiting viral infection	Anti-viral	H1N1	*J Virol* (2018)*^51^*
Ubiquitination	NS1	Blocks TRIM25-mediated RIG-I K63-Ub	Suppresses IFN-β production	Pro-viral	H1N1, H3N2	*Nat Commun* (2018)^55^
Ubiquitination	PB2	K29-Ub by CRL4^D12L1^	Non-degradative activation of polymerase	Pro-viral	H5N1, H7N9	*mBio* (2020)^49^
Ubiquitination	M2	K63-polyUb at K78 by MARCH8	Lysosomal degradation → ↓ virion egress (↓ 90% in MDCK cells)	Anti-viral	H1N1, H5N6	*Nat Commun* (2021)^53^
Ubiquitination	M1	TRIM21-mediated ubiquitination and degradation.	Restricts viral replication	Anti-viral	H1N1, H3N2, H5N1, H9N2	*Proc Natl Acad Sci U S A* (2013)^211^ *PLoS Pathog* (2023)^42^
Ubiquitination	PB1	PB1–F2 PB1–K578	Regulate the activity of viral polymerase	Pro-viral	H1N1	*PLoS One* (2015)*^212^* *Nat Commun* (2023)*^39^*
Ubiquitination	PB2	TRIM45 targets the QMRDV motif for CMA degradation.	Restricts viral replication via a non-proteasomal degradation pathway.	Anti-viral	H1N1, H3N2, H5N1, H7N9	*PLoS Pathog* (2025)^50^
Phosphorylation	PB1	Phosphorylation at S478 or T223	Disrupts polymerase function (T223 impairs RNA binding)	Anti-viral	H1N1	*PLoS Pathog* (2020)^28^
Phosphorylation	PB1	Phosphorylation at S673.	Inhibits transcription initiation but promotes replication	Pro-viral	H1N1	*PLoS Pathog* (2020)^28^
Phosphorylation	NS1	S205 phosphorylation by CK2	Recruits DDX21 to ↑ RdRp activity (↑ 300% polymerase processivity)	Pro-viral	H1N1, H3N2	*J Virol* (2021)^71^
Phosphorylation	NP	Phosphorylation at S482 by host kinase PLK3.	Facilitates NP oligomerization and vRNP assembly, promoting replication	Pro-viral	H1N1, H3N2	*Emerg Microbes Infect* (2023)^76^
Phosphorylation	M1	T108 phosphorylation	Prevents self-aggregation; enables vRNP nuclear export	Pro-viral	H1N1, H5N1	*mBio* (2023)^73^
Phosphorylation	PA	Y393	Prevents binding to the 5′-termini of viral RNA, thereby inhibiting viral polymerase function and genome replication	Anti-viral	H1N1	*Cell Reports* (2023)^69^
Phosphorylation	PB2	S181	Inhibit the replication ability and pathogenicity of highly pathogenic H5N1 virus in mice	Anti-viral	H5N1	*Virol Sin* (2024)^70^
Phosphorylation	PA	Phosphorylation at S225 in the NLS2 region	Enhances polymerase activity and vRNP assembly, increasing replication and pathogenicity of HP H5N1 in mammals	Pro-viral	H5N1	*Veterinary Microbiology* (2025)^68^
Glycosylation	NA	Conserved NLG sites on the NA head domain	Required for efficient virion incorporation and replication	Pro-viral	H1N1, H3N2	*J Virol* (2020)^91^
Glycosylation	NA	N219 glycosylation in the stalk domain	Deletion → ↓ virion budding efficiency (↓ 70%) and virulence in mice	Pro-viral	H1N1, H3N2	*J Virol* (2021)^90^
Glycosylation	HA	Glycosylation at N129 (in H5N6)	Reduces pH stability, attenuating pathogenicity and transmission in chickens	Anti-viral	H5N6	*Microbiol Spectr* (2022)^84^
Glycosylation	HA	Glycosylation at position 127 in vaccine strains.	Improves protection against H9N2 while reducing reassortant virus virulence	Anti-viral	H9N2	*Transbound Emerg Dis* (2022)^97^
Glycosylation	HA	D158N/N193K mutations in RBD	Dual α2-3/α2-6 sialic acid binding → bovine mammary/human conjunctiva tropism	Pro-viral	Clade 2.3.4.4b H5N1	*Lancet Microbe* (2025)^85^
Glycosylation	HA	Gain or alteration of glycosylation sites on the HA globular head.	Shields antigenic epitopes to facilitate immune evasion	Pro-viral	H13 subtypes	*Virology* (2025)^86^
SUMOylation	NP	Multiple potential loci (such as K7, K48, K87	Promote virus replication	Pro-viral	H5N1	*J Virol* (2014)^100^ *Front Microbiol* (2020)^213^
SUMOylation	PB1	K612 SUMOylation	↑ vRNA binding affinity; essential for ferret respiratory transmission	Pro-viral	H1N1, H3N2	*PLoS Pathog* (2021)^100^^,^^101^
SUMOylation	M1	SUMOylation at residue A215 and filamentous morphology associated with D30.	Enhance replication and virulence in mammalian hosts	Pro-viral	H5N1	*J. Virol.* (2011)^106^ *J. Virol.* (2022)^105^
SUMOylation	PB2	The SUMO E3 ligase activity of PIAS1 catalyzed robust SUMOylation of PB2	Inhibit the activity of the viral RNP complex and viral replication	Anti-viral	H1N1	*PLoS Pathog* (2022)^104^
Acetylation	NP	K229 acetylation	Influence the activity of viral polymerase	Regulatory	H1N1, H5N1	*Nat Commun* (2017)^111^ *Front Immunol* (2017)^112^ *J Biol Chem* (2018)^214^
Acetylation	PA-X (and PA)	NatB complex acetylates the N-terminal Met–Glu start	Required for host shutoff activity and viral polymerase function	Pro-viral	H1N1, H3N2, H5N1, H7N9	*Cell Reports* (2018)^114^
Acetylation	NS1	K108 acetylation	Modulates innate immune responses.	Pro-viral	H1N1	*Veterinary Research* (2020)^115^
Acetylation	PA	K674 acetylation by PCAF	↑ Endonuclease activity → ↑ viral mRNA cleavage	Pro-viral	H3N2, H7N9	*FEBS J* (2022)^113^
Acetylation	HA	K157, K169, K418, K459	The acetylation of K157 and K418 is crucial to the virulence of the virus	Pro-viral	HIN1	*Emerg Microbes Infect* (2026)^116^
Carboxylation	HA	GGCX carboxylates HA at residue E225	Promotes virus binding to human-type (α-2,6 SA) receptor	Pro-viral	H1N1	*Nature Communications* (2025)^121^
Palmitoylation	M2	Palmitoylation modification sites exist on the amphiphilic helix near the transmembrane region of the M2 protein	Palmitoylation guides M2 to be located on lipid rafts	Pro-viral	H1N1	*Biochem J* (2011)^129^ *Sci Rep* (2023)^130^
Palmitoylation	HA	ER-localized DHHC enzymes 1, 2, 4, and 6 catalyze HA palmitoylation	Required for efficient virus replication	Pro-viral	Influenza B virus	*Journal of Virology* (2023)^127^
Proteolytic cleavage	M2	Caspase cleavage at the 82SAVD85 motif	Abolishes M2-LC3 interaction, reducing virion production	Anti-viral	H3, H5, H9 subtype influenza A viruses	*EMBO Rep* (2025)^136^

### Ubiquitination: A spatiotemporal determinant of viral fate

Ubiquitin is a small, evolutionarily conserved eukaryotic protein composed of 76 amino acids containing seven lysine residues (K6, K11, K27, K29, K33, K48, K63) and an N-terminal methionine.^33^^,^^34^ Ubiquitination is an enzymatic process involving the covalent attachment of one or more ubiquitin molecules to target proteins,^35^ mediated by a cascade of ubiquitin-activating (E1), conjugating (E2), and ligating (E3) enzymes.^36^, ^37^, ^38^ Ubiquitination serves as a master regulator across multiple stages of the viral life cycle in Figure 3. In influenza A virus, ubiquitination plays a critical regulatory role in viral protein function and host–pathogen interactions, and is indispensable for efficient viral replication.^39^^,^^40^

During the early stages of infection, encompassing viral entry and uncoating (Fig. 3, steps 1 & 2), ubiquitination immediately influences the fate of incoming virions.^41^ The host deploys it as a defensive weapon: for instance, the E3 ligase TRIM21 directly ubiquitinates the viral matrix protein M1, targeting it for proteasomal degradation to abort early replication.^42^ Conversely, the virus co-opts this modification for its own purposes, as evidenced by the essential role of M2 protein ubiquitination at lysine 78 in promoting subsequent genomic RNA packaging.^43^ This early tug-of-war sets the tone for a replication cycle heavily influenced by ubiquitin dynamics.^44^

As the viral replication cycle progresses to its mid-phase, which focuses on genome replication and particle assembly (Fig. 3, steps 4 & 5), ubiquitination predominantly assumes proviral functions to stabilize and coordinate the viral machinery.^45^, ^46^, ^47^ carbon catabolite repression 4 (Ccr4)–negative on TATA-less (Not) transcription complex subunit 4 (CNOT4)-mediated ubiquitination of NP enhances vRNA replication,^48^ while specific E3 ligases like those in the cullin-RING ligase 4 (CRL4) complex enhance PB2 polymerase activity via ubiquitination.^49^ Host defenses, however, remain vigilant. A recent frontier discovery highlights tripartite motif-containing 45 (TRIM45), which restricts infection by orchestrating the chaperone-mediated autophagic degradation of PB2, revealing a novel, ubiquitin-dependent antiviral pathway targeting the core replication complex.^50^ Similarly, the E3 ubiquitin ligase TRIM41 directly polyubiquitinates NP, leading to its degradation via the proteasome pathway and thereby limiting viral replication.^51^ However, K63-linked ubiquitination at K102 of M1 emerges as a critical positive signal driving viral egress.^52^

In the late stages of infection, involving virion release and the ongoing battle with host immunity (Fig. 3, step 6), ubiquitination continues to fine-tune the infectious outcome. While it facilitates late-budding processes, it also remains a target of host restriction factors such as membrane-associated Ring–CH–type finger 8 (MARCH8), which directs M2 toward lysosomal degradation.^53^ Beyond physical virus assembly, ubiquitination is central to the immune signaling interface.^54^ The viral NS1 protein exemplifies sophisticated interference, directly binding to and inhibiting the ubiquitin-activated antiviral protein TRIM25, a mechanism whose structural basis has been recently elucidated.^55^ This strategic disruption of host ubiquitin pathways underscores its critical role in immune evasion.^56^^,^^57^

### Phosphorylation: Fine-tuning viral replication through conformational and functional switches

Figure 1 highlights phosphorylation as a major modification of PA, PB1, PB2, and NP, implicating it directly in the core processes of transcription, replication, and RNP dynamics.^58^^,^^59^ Phosphorylation involves the enzymatic transfer of γ-phosphate groups from nucleotides such as ATP or GTP to hydroxyl groups on serine (Ser), threonine (Thr), or tyrosine (Tyr) residues of target proteins, catalyzed by specific protein kinases.^60^^,^^61^ Phosphorylation and dephosphorylation modifications function as exquisitely coordinated regulatory elements,^62^ constituting a pivotal molecular rheostat that fine-tunes influenza virus replication efficiency.^63^^,^^64^ Phosphorylation operates as a dynamic and reversible molecular switch, fine-tuning the function of viral and host proteins at decisive points throughout the influenza life cycle (Fig. 3).^65^^,^^66^

A primary focus of phosphorylation is the precise regulation of the viral polymerase complex and genome replication fidelity (Fig. 3, step 4). This modification acts as a sophisticated control panel for the RNA-dependent RNA polymerase (RdRp).^67^ Phosphorylation of PB1 at Ser673, for example, provides a critical functional switch, selectively inhibiting viral transcription initiation while promoting genome replication, thereby modulating the balance between these two essential modes.^28^ Recent evidence extends this regulatory paradigm to virulence determinants, as phosphorylation of PA at Ser225 has been shown to enhance the fitness of highly pathogenic H5N1 avian influenza virus in mammalian hosts, underscoring how site-specific phosphorylation can directly influence viral adaptation and pathogenesis.^68^ In striking contrast, phosphorylation at Tyr393 of PA impairs polymerase function by preventing RNA binding.^69^ Research has found that phosphorylation at the S181 site of PB2 significantly inhibits the replication ability and pathogenicity of highly pathogenic H5N1 virus in mice.^70^ Beyond the polymerase subunits themselves, phosphorylation also indirectly potentiates replication; cellular kinase CK2-mediated phosphorylation of the NS1 protein at Ser205 enhances its interaction with the host factor DExD-box helicase 21 (DDX21), leading to increased viral polymerase activity.^71^

The intracellular journey of vRNP complexes, particularly their nuclear export (Fig. 3, step 3), is another key process governed by phosphorylation. The matrix protein M1 serves as a central effector in this transport.^72^ Phosphorylation at Thr108 of M1 is essential, as it prevents premature self-aggregation at the plasma membrane.^73^ The non-phosphorylated form of M1 mislocalizes and aggregates, severely impairing its crucial role in mediating vRNP export from the nucleus.^73^^,^^74^ This precise phosphorylation-dependent conformation change ensures the timely and orderly progression of vRNPs to the budding site.^75^

During the assembly phase (Fig. 3, step 5), phosphorylation contributes to the structural organization of viral components. Phosphorylation of NP at Ser482 facilitates its oligomerization, promoting the proper assembly of vRNPs, which are the core genetic packages incorporated into new virions.^76^ Conversely, phosphorylation at a different site, such as Ser165 of NP, can disrupt oligomerization and impair RNP activity, illustrating how the same PTM type can exert opposite effects depending on the specific residue modified, allowing for exquisite layered control.^77^ Host kinases also participate in this regulatory network, with factors like c-Jun NH2-terminal kinase (JNK)-interacting protein 4 (JIP4) exhibiting antiviral effects through phosphorylation-dependent mechanisms that disrupt essential viral processes.^78^

### Glycosylation: The surface glycan shield governing viral entry and exit

Glycosylation, predominantly as N-linked glycosylation (NLG) on the surface glycoproteins HA and NA (Fig. 1), constitutes the primary interface between the influenza virion and its host.^79^ Its functions are strategically localized to the bookends of the viral life cycle (Fig. 3), critically determining the success of initial cellular attachment and final release while continuously negotiating immune recognition.^80^^,^^81^

The critical first step of viral attachment and cellular entry (Fig. 3, step 1) is governed by the glycan landscape on HA. Glycans attached to specific asparagine residues on the HA globular head directly modulate receptor-binding avidity and specificity by influencing the conformation of the receptor-binding site.^82^^,^^83^ The presence or absence of a glycan at a key position, such as N129 in H5N6 viruses, can alter receptor-binding properties, thereby affecting viral infectivity and host range.^84^ Recent high-resolution structural studies have powerfully illustrated this principle. Research on cattle-infecting H5N1 viruses demonstrated that specific glycosylation patterns, alongside amino acid changes, are integral to shaping the receptor-binding site for optimal engagement with host receptors in new species.^85^ Similarly, work on gull-adapted H13 viruses directly links unique glycosylation-mediated receptor-binding characteristics to their distinct host preferences.^86^ These findings underscore that HA glycosylation is a dynamic determinant of interspecies transmission and tissue tropism.^87^

At the culmination of the replication cycle, glycosylation is equally pivotal for efficient virion release and for evading pre-existing host immunity (Fig. 3, steps 5 & 6).^88^, ^89^, ^90^ For NA, glycosylation is not merely decorative but essential for its enzymatic function.^91^^,^^92^ NLG on the NA head domain, particularly at conserved sites, is required for proper protein folding, stability, and efficient incorporation into budding virions.^91^^,^^93^ Loss of these glycans impairs NA's sialidase activity, leading to severe defects in viral replication and release, as demonstrated for residues critical for NA structure.^90^^,^^91^ Concurrently, the dense array of glycans on HA forms a physical “glycan shield” that sterically blocks antibody access to underlying protein epitopes.^94^^,^^95^ This shielding is a major driver of antigenic drift, as the acquisition of new glycosylation sites can mask antigenic regions, allowing the virus to escape neutralization while compensating for potential fitness costs associated with receptor-binding changes.^96^ The strategic engineering of glycosylation patterns, such as modifying site 127 in vaccine strains, has proven effective in modulating immunogenicity and protective efficacy, highlighting its direct translational relevance.^97^ Thus, glycosylation masterfully balances the conflicting demands of receptor engagement, enzymatic release, and perpetual immune evasion.

### SUMOylation: Molecular conductor directing viral trafficking and assembly with precision

SUMOylation serves as a pivotal post-translational regulatory mechanism in influenza virus pathogenesis, where the virus hijacks the host enzymatic machinery to precisely modify target proteins, thereby orchestrating viral replication, assembly, and immune evasion through spatiotemporal regulation, as delineated in Figure 2.^98^^,^^99^ This spatial and temporal precision is best understood through a stage-specific lens. During the initial nuclear phase of genome replication and transcription (Fig. 2), PIASx-mediated SUMOylation of NP at K4/K7 is essential for its nuclear localization and vRNP assembly,^100^ while concurrent SUMOylation of PB1 at K612 stabilizes the polymerase complex by enhancing its vRNA-binding affinity.^101^ Notably, the viral NS2 protein exploits this system via its SIM to bind to SUMOylated host proteins huANP32 A/B, enhancing polymerase activity,^102^^,^^103^ whereas the host counteracts via protein inhibitor of activated STAT 1 (PIAS1)-mediated SUMOylation of PB2 to restrict replication.^104^

In the late stage of the viral life cycle, the role of SUMOylation shifts accordingly, as illustrated in Figure 2. At the cytoplasmic membrane, SUMOylation of M1 at K242 is indispensable for viral matrix layer formation and virion morphogenesis.^105^^,^^106^ Before this, efficient vRNP export from the nucleus is facilitated by HA, which triggers a switch from SUMOylation to phosphorylation of the host kinase MAP kinase kinase 1 (MEK1), thereby activating the mitogen-activated protein kinase (MAPK)/extracellular signal-regulated kinase (ERK) export pathway.^107^

Concurrently, SUMOylation is co-opted to suppress host antiviral defenses throughout the infection cycle. SUMOylation of NS1, notably at K219, enhances its stability and intensifies its suppression of the host interferon response, such as blocking interferon beta (IFN-β) promoter activity.^108^ In summary, SUMOylation acts as a molecular conductor, providing the stage-specific and spatially resolved regulatory cues essential for viral progression, a coordinated interplay between viral components and host pathways that is comprehensively summarized in Figure 2.

### Emerging PTMs: expanding the regulatory frontier

Beyond the major PTMs cataloged in Figure 1, recent years have unveiled a spectrum of emerging modifications that expand the regulatory lexicon of influenza–host interactions. These include acetylation, methylation, and novel types like carboxylation, which often target HA (as hinted in the evolving landscape of Figure 1). This section will highlight these frontier discoveries, emphasizing how they reveal unexpected mechanisms of viral adaptation and host defense.

Acetylation has been solidified as a major regulator, with viruses strategically manipulating host acetyltransferases and deacetylases.^109^^,^^110^ This modification fine-tunes viral protein function and subverts innate immunity at multiple stages. For instance, acetylation of the NP modulates its function in viral transcription and replication,^111^^,^^112^ while acetylation of the PA polymerase subunit enhances its endonuclease activity, critical for the viral “cap-snatching” mechanism during transcription initiation.^113^ Recent studies highlight the importance of site-specific acetylation on non-structural proteins: N-terminal acetylation of PA-X by host NatB is essential for its host shutoff activity, a key virulence function,^114^ and acetylation at lysine 108 of the NS1 protein significantly contributes to viral replication and pathogenesis.^115^ A recent study published in 2026 first identified acetylation sites on the H1N1 viral HA protein, with acetylation at K157 and K418 being crucial for viral virulence.^116^ Furthermore, the virus exploits host deacetylases like histone deacetylase 6 (HDAC6) to deacetylate and inhibit the innate immune sensor retinoic acid-inducible gene I (RIG-I), thereby blunting the interferon response.^117^^,^^118^

Carboxylation, a relatively new player in virology, has been implicated in direct viral adaptation.^119^^,^^120^ A paradigm-shifting study revealed that the host enzyme γ-glutamyl carboxylase (GGCX) can carboxylate the viral HA.^121^ This modification alters HA's receptor-binding preference, effectively steering the virus towards human-type receptors and facilitating cross-species adaptation.^121^ This discovery provides a striking example of how host biochemistry can directly shape viral evolution and tropism.

Lipid metabolism orchestrates multiple stages of influenza A virus replication, with distinct lipid species exerting divergent effects.^122^, ^123^, ^124^ Comprehensive analyses confirm that the metabolism of fatty acids, cholesterol, phospholipids, and glycolipids is extensively reprogrammed upon infection and may either suppress or promote influenza A virus outcome.^122^^,^^123^ For instance, cholesterol metabolism has been shown to facilitate viral entry and replication,^124^^,^^125^ while specific plasma membrane lipid compositions can restrict infection.^126^ Notably, palmitoylation is indispensable for influenza B virus replication.^127^ The palmitoylation of HA is a signal associated with membrane rafts.^128^ The palmitoylation of the M2 protein, achieved through the cooperation of its N-terminal amphipathic helix and transmembrane domain, is essential for its accurate localization to the plasma membrane.^129^^,^^130^ While lipid metabolism represents a promising therapeutic target for anti-influenza drug development, current evidence remains descriptive, with underlying mechanisms yet to be fully elucidated.^127^^,^^131^

Regulated proteolytic cleavage beyond canonical processing is emerging as a crucial regulatory PTM.^132^, ^133^, ^134^, ^135^ A landmark 2025 study identified that the influenza A virus M2 protein is cleaved by host caspase enzymes during infection.^136^ This cleavage event disrupts the interaction between M2 and the autophagy protein light chain 3 (LC3), and finely regulates the production of infectious viral particles.^136^ This finding introduces caspase-mediated cleavage as a novel, host-mediated PTM that critically controls the late stages of virion assembly and release, opening a new avenue for understanding how programmed cell death pathways intersect with viral replication cycles.

ISGylation is an interferon-stimulated gene (ISG15)-mediated host ubiquitin-like modification and serves as a crucial antiviral defense mechanism.^137^ In influenza viruses, this modification primarily targets proteins such as NS1 and NP to inhibit viral replication (Fig. 1)^138^^,^^139^; the viruses, in turn, employ strategies like suppressing the interferon response or directly sequestering ISGylated proteins to evade this host defense.^139^

### The multilayered crosstalk network of PTMs in influenza virus infection

While individual PTM types regulate specific stages of the infection cycle, they function not in isolation but as components of a dynamic, multilayered network.^140^ Extensive cross-talk between modifications adds a critical layer of control, forming a precise molecular battleground that determines infection outcome,^141^ as synthesized in Figure 4.

### Host PTM networks dominate influenza biology

As shown in Figure 4, the influenza virus is highly dependent on host PTMs to achieve replication, immune evasion, and assembly.^142^ Modifications include ubiquitination, phosphorylation, glycosylation, SUMOylation, and other emerging modifications, forming a multilayered, interwoven network that regulates viral components and host antiviral signaling, inflammatory responses, and cell fate, thereby determining replication kinetics and disease progression.^8^^,^^19^

### Key modifications and their interactive crosstalk

Phosphorylation and ubiquitination constitute the central axes, engaging in intricate cross-talk with other PTMs.^8^^,^^30^^,^^143^ Phosphorylation acts as a switch, modulating vRNP activity, nuclear import, and transcription; ubiquitination exerts bidirectional effects through degradation, signaling, and polymerase regulation, and is exploited by the virus to promote uncoating and assembly.^8^^,^^39^^,^^144^^,^^145^ SUMOylation modifications do not occur in isolation; instead, they engage in complex cross-talk with acetylation, phosphorylation, and others to co-regulate protein function.^146^^,^^147^ Representative interactions include NP Y78 phosphorylation inhibiting ubiquitination (Fig. 4), thereby impacting nuclear export and replication timing;^143^ PB1 SUMOylation critical for stability and polymerase activity;^101^ and modification cascades on PB2/PA potentially triggering downstream degradation.^50^^,^^148^^,^^149^ Metabolic–PTM cross-talk also emerges along this axis, exemplified by GGCX-mediated MAVS carboxylation and lactate-induced lactylation that synergistically shape antiviral signaling, broadening our understanding of cross–modification interactions.^119^^,^^150^^,^^151^

### Therapeutic potential of targeting the PTM network

As illustrated in Figure 4, influenza viruses hijack and remodel host PTM networks by exploiting regulatory nodes of host E3 ubiquitin ligases (*e.g.*, specific TRIM family members) and deubiquitinases (*e.g.*, OTUB1) to fine-tune antiviral signaling (*e.g.*, MAVS) and the stability, assembly, and immune evasion of viral proteins.^50^^,^^152^, ^153^, ^154^, ^155^ Host glycosylation and receptor modifications, inclusion-body maturation, and PML nuclear-body mediated defenses are likewise sculpted by the PTM landscape.^156^, ^157^, ^158^, ^159^

### Clinical transformation potential and challenges

#### Development of treatment strategies

Small-molecule inhibitors targeting PTMs represent a promising class of antiviral agents.^160^^,^^161^ These inhibitors are characterized by low molecular weight and excellent cell permeability, disrupt viral protein PTM processes, such as phosphorylation, acetylation, or ubiquitination, to impair viral replication and pathogenesis.^162^^,^^163^ Their pharmacological advantages, including favorable pharmacokinetic profiles, position them as critical candidates in antiviral drug development.^164^^,^^165^ For example, R406, a spleen tyrosine kinase (SYK) inhibitor, competitively blocks ATP binding to inhibit SYK autophosphorylation at Tyr525/526, which in turn suppresses SYK-mediated signaling cascades, thereby attenuating viral replication and ameliorating influenza-associated lung pathology.^166^ Natural product-derived compounds, such as 3-Anhydro-6-hydroxy-ophiobolin A, which demonstrates high efficacy against influenza A virus both *in vitro* and *in vivo*, offer promising lead structures for novel antiviral development by targeting virus–host interactions.^167^

These small-molecule inhibitors competitively target substrate-binding sites—exemplified by EZH2 inhibitors blocking histone H3K27 methylation to suppress tumor epigenetic reprogramming—and cofactor-binding pockets, as demonstrated by PRMT5 inhibitors disrupting arginine methylation through SAM-binding pocket occupancy.^168^ Furthermore, novel bifunctional agents such as PhosTACs (a concept advanced post-2020) recruit phosphatases to catalytically dephosphorylate target proteins in a suprastoichiometric manner, overcoming traditional occupancy-driven resistance.^169^ Future investigations should expand toward novel PTM targets and explore PTM crosstalk, including phosphorylation–ubiquitination interplay via proteolysis targeting chimera (PROTAC)-mediated degradation of drug-resistant proteins.^170^

#### Vaccine design insights

PTMs offer novel perspectives in influenza vaccine development.^171^ Identification of PTM sites and their functional roles enables the design of modified antigens with improved immunogenicity.^8^ Beyond static modifications, emerging strategies exploit dynamic PTM pathways for antigen engineering. For instance, the PROTAR 2.0 platform introduces regulatory proteolytic tags into viral proteins, harnessing the host ubiquitin–proteasome system to degrade antigens in a controlled manner and thereby enhance vaccine efficacy—exemplifying how regulated proteolysis, as a crucial PTM, can be co-opted for immunization.^132^ Concurrently, glycosylation of influenza viral proteins modulates their antigenicity and immunogenicity; strategic NLG engineering of HA balances viral infectivity with vaccine immunogenicity.^172^^,^^173^ Optimizing glycosylation profiles and expression systems may further advance vaccine potency.^174^ Influenza vaccine design requires quantifying HA strain similarity through antigenic cartography to correlate protein sequences with antigenicity, leveraging glycosylation similarity among related viral strains provides critical criteria for selecting efficacious vaccine candidates.^175^ Future efforts should focus on novel vaccine technologies (*e.g.*, mRNA platforms) to achieve efficient delivery and robust expression of PTM-optimized viral antigens.

#### Current challenges

During replication, viruses hijack host PTM machinery to refine viral protein functions, enhancing infectivity or evading immune surveillance.^18^^,^^176^ Over 700 PTM types have been identified in virus–host interactions, with influenza viral proteins exhibiting diverse modifications, including phosphorylation, ubiquitination, acetylation, methylation, and glycosylation in Figure 1.^19^^,^^177^ These modifications exert distinct effects on protein behavior, and individual proteins may harbor multiple modification sites, with individual residues undergoing distinct modifications under varying spatiotemporal conditions.^8^^,^^178^ As we have traversed from viral attachment to immune evasion, Figure 3 has served as our guiding map, illustrating how PTMs sequentially orchestrate each step of the influenza life cycle. This complexity poses significant challenges in elucidating the dynamic interplay of PTMs. For example, lysine residues K77, K113, and K229 on NP undergo acetylation, where K229 modification modulates viral particle release, whereas K113 acetylation-mimetic mutations compromise viral growth, and simultaneous K77/K229 acetylation-mimetic mutations synergistically impair viral replication.^111^ However, comprehensively mapping the spatiotemporal dynamics of these modifications during infection remains experimentally demanding.

Viral proteins engage in extensive interactions with host cellular proteins, forming intricate networks critical for viral replication, transcription, assembly, and immune evasion.^8^^,^^178^^,^^179^ These interactions are regulated by host PTM systems, while host cells simultaneously employ counteractive mechanisms to disrupt viral protein functionality.^180^^,^^181^ This intricate network of interactions complicates the dissection of the spatiotemporal dynamics governing virus–host PTM interplay. Influenza NS1 protein interacts with host RIG-I to induce conformational alterations that suppress RIG-I activation, thereby subverting antiviral responses; while NS1 employs multifaceted mechanisms—including protein interactions, ubiquitination interference, and cytokine/IFN suppression—to inhibit host defenses, a comprehensive understanding of this dynamic interplay across infection stages and cellular contexts necessitates integrative analyses.^55^^,^^182^ Currently, while studies directly comparing the global PTM differences among different influenza virus subtypes are limited, clear evidence indicates significant variations in key biological phenotypes (*e.g.*, antigenic evolution) and dependency on PTMs (*e.g.*, HA glycosylation) across subtypes.^8^^,^^183^^,^^184^ These differences strongly suggest that subtype-specific heterogeneity in PTM patterns may be an underappreciated, yet clinically critical feature affecting vaccine efficacy and viral pathogenicity.^183^^,^^184^ This heterogeneity manifests not only in differential modification sites and stoichiometry across influenza A virus subtypes but also in the functional consequences for viral-host signaling crosstalk, directly impacting pathogenesis.^185^, ^186^, ^187^ For instance, while pandemic (*e.g.*, avian H5N1) and seasonal (*e.g.*, H1N1, H3N2) strains may share core strategies like NS1-mediated immune antagonism, subtype-specific sequence variations can alter PTM profiles, interaction interfaces, and ultimately viral fitness within a host population.^19^ Monitoring the evolution of these PTM-associated sequences over time offers a valuable lens through which to view viral adaptation and zoonotic potential, highlighting the direct translational importance of PTM research for pandemic preparedness and antiviral design.^188^

Current methodologies for investigating viral protein PTMs primarily include mass spectrometry, gene editing, and cellular assays.^189^, ^190^, ^191^ The advantages and limitations of these techniques in resolving the complex, heterogeneous nature of viral PTMs are summarized in Table 2. While each technique has distinct advantages and limitations in resolving the heterogeneity of modification patterns across subtypes, their synergistic and combined application is indispensable for generating comprehensive insights.^192^, ^193^, ^194^ For example, mass spectrometry identifies a novel phosphorylation site on NP, CRISPR engineering creates an isogenic virus with a phospho-ablation mutation, and cellular assays then reveal its specific defect in viral genome packaging.^59^^,^^143^^,^^195^^,^^196^ However, the sheer complexity, dynamism, and context-dependency of viral PTM networks still present multidimensional challenges that existing technologies cannot fully address.^197^, ^198^, ^199^

**Table 2:** Advantages and limitations of core methodologies for studying post-translational modifications (PTMs) of influenza virus proteins.

Method	Key advantages	Major limitations
Mass spectrometry	Unbiased discovery: Enables global, hypothesis-free mapping of PTM sites Quantitative precision: Provides stoichiometric and temporal data on PTM dynamics	Functional blind spot: Identifies modification sites but does not directly reveal biological function Sensitivity constraint: Struggles with low-abundance or transient modifications
Gene editing (*e.g.*, CRISPR-Cas)	Direct functional validation: Enables causal linkage between specific PTM sites and viral function High-throughput screening: can systematically identify host factors regulating PTMs	Technical complexity: Requires expertise in the delivery and analysis of relevant models Off-target effects: risk of unintended genomic alterations
Cellular & biochemical assays	Direct functional readout: Measures concrete impact on protein activity, localization, or interactions Physiological context: can be performed in authentic infection models	Hypothesis-driven: Requires prior knowledge, limiting discovery potential Low throughput: Makes systematic study across multiple PTMs labor-intensive

## Conclusion

PTMs orchestrate influenza virus pathogenesis: HA glycosylation dictates receptor specificity during attachment,^200^ while M2 phosphorylation drives endosomal acidification for vRNP liberation via HA-mediated fusion.^25^ Nuclear trafficking is accelerated by NP SUMOylation-enhanced biomolecular condensation and dynein phosphorylation.^201^ M1 palmitoylation enables lipid-raft-dependent budding through membrane curvature induction,^200^^,^^201^ while NA glycosylation optimizes release efficiency, and HA O-GlcNAcylation/NS1-mediated interferon blockade executes immune evasion^25^; the relevant processes are shown in Figure 3.

Recent research highlights extensive cross-talk between distinct PTM layers. Targeting these nodes holds transformative potential: R406 inhibits SYK autophosphorylation to attenuate lung pathology, Anemoside B4 stabilizes MAVS signalosomes via enhanced UFMylation, and PhosTACs catalytically dephosphorylate drug-resistant viral targets.^166^^,^^169^^,^^202^ Yet fundamental challenges persist in resolving spatiotemporal PTM dynamics across infection stages and viral subtypes, necessitating advanced methodologies integrating cryo-ET with single-cell modificomics.^203^^,^^204^ Future antiviral design must leverage combinatorial targeting of PTM enzyme networks (*e.g.*, PIAS1–PB2 SUMOylation) while exploiting glycoengineering to refine vaccine antigens.^117^^,^^205^ Deciphering this multidimensional PTM landscape will illuminate universal vulnerabilities across influenza variants and accelerate next-generation therapeutics.

## CRediT authorship contribution statement

**Menghuan Cui:** Writing – review & editing, Writing – original draft, Visualization, Validation, Investigation, Formal analysis, Data curation. **Shuyi Wang:** Writing – review & editing, Validation, Methodology, Data curation. **Xiao Fei:** Writing – review & editing, Validation, Supervision, Project administration, Formal analysis, Data curation. **Guijie Guo:** Writing – review & editing, Supervision, Resources, Project administration, Funding acquisition, Conceptualization.

## Funding

This work was supported by the 10.13039/501100001809National Natural Science Foundation of China (No. U25A20713, 32372998).

## Conflict of interests

The authors declared no conflict of interests.
